# Uncommon nucleotide excision repair phenotypes revealed by targeted high-throughput sequencing

**DOI:** 10.1186/s13023-016-0408-0

**Published:** 2016-03-22

**Authors:** Nadège Calmels, Géraldine Greff, Cathy Obringer, Nadine Kempf, Claire Gasnier, Julien Tarabeux, Marguerite Miguet, Geneviève Baujat, Didier Bessis, Patricia Bretones, Anne Cavau, Béatrice Digeon, Martine Doco-Fenzy, Bérénice Doray, François Feillet, Jesus Gardeazabal, Blanca Gener, Sophie Julia, Isabel Llano-Rivas, Artur Mazur, Caroline Michot, Florence Renaldo-Robin, Massimiliano Rossi, Pascal Sabouraud, Boris Keren, Christel Depienne, Jean Muller, Jean-Louis Mandel, Vincent Laugel

**Affiliations:** Laboratoire de Diagnostic Génétique, Institut de Génétique Médicale d’Alsace (IGMA), Hôpitaux Universitaires de Strasbourg, 1 place de l’hôpital, Strasbourg, France; Laboratoire de Génétique Médicale – INSERM U1112, Institut de Génétique Médicale d’Alsace (IGMA), Faculté de médecine de Strasbourg, 11 rue Humann, Strasbourg, France; Centre de Référence Maladies Osseuses Constitutionnelles, Département de Génétique, Hôpital Necker-Enfants malades, Paris, France; Département de Dermatologie, Hôpital Saint-Éloi, 80 avenue Augustin-Fliche, 34295 Montpellier, France; Service d’Endocrinologie Pédiatrique, diabète et maladies héréditaires du métabolisme, Hôpital Femme Mère enfant, GH Est, 59 boulevard Pinel, Bron, France; Service de Pédiatrie Générale, Hôpital Necker-Enfants malades, Paris, France; Service de Pédiatrie, CHU de Reims, Hôpital Maison Blanche, 45 rue Cognacq-Jay, Reims, France; Service de Génétique et Biologie de la Reproduction CHU de Reims, Hôpital Maison Blanche, 45 rue Cognacq-Jay, Reims, France; Service de Génétique, CHU La Réunion, Hôpital Félix Guyon, Allée des Topazes, Saint-Denis, France; Centre de Référence des Maladies Héréditaires du Métabolisme, Service de Médecine Infantile, INSERM NGERE 954, CHU Brabois Enfants, Allée du Morvan, Vandœuvre les Nancy, France; Servicio de Dermatología, Cruces University Hospital, BioCruces Health Research Institute, Baracaldo Vizcaya, Spain; Servicio de Genética, Cruces University Hospital, BioCruces Health Research Institute, Baracaldo Vizcaya, Spain; Service de Génétique Médicale, CHU de Toulouse - Hôpital Purpan, Place du Docteur Baylac, Toulouse, France; Department of Pediatrics, Pediatric Endocrinology and Diabetes, Faculty of Medicine, University of Rzeszów, Rzeszów, Poland; Service de Génétique Médicale, Hôpital Necker Enfants-Malades, 24 Bd du Montparnasse, Paris, France; Service de Neurologie, Hôpital Robert Debré, 48 boulevard Serurier, Paris, France; Centre de Référence des Anomalies du Développement, Service de Génétique, Hospices Civils de Lyon, Lyon, France; INSERM U1028; CNRS UMR5292; CNRL TIGER Team, Lyon, France; Service de Pédiatrie A - Neurologie pédiatrique, CHU de Reims - American Memorial Hospital, 47 rue Cognacq Jay, Reims, France; AP-HP, Hôpital de la Pitié-Salpêtrière, Département de Génétique, F-75013 Paris, France; Sorbonne Universités, UPMC Univ Paris 06, Inserm, CNRS, UM 75, U 1127, UMR 7225, ICM, F-75013, Paris, France; Service de Pédiatrie, Hôpitaux Universitaires de Strasbourg, 1 avenue Molière, Strasbourg, France

**Keywords:** NER, NGS, Cockayne syndrome, xeroderma pigmentosum, *ERCC2*, *ERCC3*, *ERCC5*, *ERCC6*, *ERCC8*, *POLH*

## Abstract

**Background:**

Deficient nucleotide excision repair (NER) activity causes a variety of autosomal recessive diseases including xeroderma pigmentosum (XP) a disorder which pre-disposes to skin cancer, and the severe multisystem condition known as Cockayne syndrome (CS). In view of the clinical overlap between NER-related disorders, as well as the existence of multiple phenotypes and the numerous genes involved, we developed a new diagnostic approach based on the enrichment of 16 NER-related genes by multiplex amplification coupled with next-generation sequencing (NGS).

**Methods:**

Our test cohort consisted of 11 DNA samples, all with known mutations and/or non pathogenic SNPs in two of the tested genes. We then used the same technique to analyse samples from a prospective cohort of 40 patients. Multiplex amplification and sequencing were performed using AmpliSeq protocol on the Ion Torrent PGM (Life Technologies).

**Results:**

We identified causative mutations in 17 out of the 40 patients (43 %). Four patients showed biallelic mutations in the *ERCC6(CSB)* gene, five in the *ERCC8(CSA)* gene: most of them had classical CS features but some had very mild and incomplete phenotypes. A small cohort of 4 unrelated classic XP patients from the Basque country (Northern Spain) revealed a common splicing mutation in *POLH* (XP-variant), demonstrating a new founder effect in this population. Interestingly, our results also found *ERCC2(XPD), ERCC3(XPB)* or *ERCC5(XPG)* mutations in two cases of UV-sensitive syndrome and in two cases with mixed XP/CS phenotypes.

**Conclusions:**

Our study confirms that NGS is an efficient technique for the analysis of NER-related disorders on a molecular level. It is particularly useful for phenotypes with combined features or unusually mild symptoms. Targeted NGS used in conjunction with DNA repair functional tests and precise clinical evaluation permits rapid and cost-effective diagnosis in patients with NER-defects.

**Electronic supplementary material:**

The online version of this article (doi:10.1186/s13023-016-0408-0) contains supplementary material, which is available to authorized users.

## Background

Nucleotide excision repair (NER) is a multistep DNA repair process in which a broad spectrum of DNA lesions are removed in two stages - one acting genome-wide (GG-NER) and the other on actively transcribed strands (TC-NER) [1]. Despite a common autosomal recessive pattern of inheritance, genetic defects in the NER pathway lead to a broad variety of clinical conditions, namely: xeroderma pigmentosum (XP), trichothiodystrophy (TTD), Cockayne syndrome (CS) and UV-sensitive syndrome (UVSS) (Table 1). **Xeroderma pigmentosum** (Orpha number 910) is a genodermatosis characterized by extreme sensitivity to ultraviolet (UV)-induced changes in the skin and eyes, and multiple skin cancers. XP patients present sunlight-induced pigmentation changes, a predisposition to skin cancer and in about 30 % of cases neurological degeneration with progressive cognitive impairment. Classical XP results from defects in one of seven genes, *XPA* through to *G*. XP-variant, characterized by the absence of DNA repair deficits, results from a deficiency in the translesion synthesis DNA polymerase, polη [2]. **Trichothiodystrophy** (Orpha number 33364) is a very rare heterogeneous group of disorders characterized by brittle hair with low-sulfur content associated with neurodevelopmental impairment. The spectrum of symptoms observed in TTD patients ranges from mild forms of the disease, characterized by normal development with brittle hair and scaling skin only, to very severe cases, characterized by high mortality at a young age combined with severe neurodevelopmental defects. Approximately 50 % of patients with TTD exhibit marked photosensitivity. These photosensitive patients are found to be carriers of biallelic mutations, usually in the *ERCC2(XPD)* gene, or more rarely in *ERCC3(XPB)* or *TTDA* [3]. All encode for subunits of the dual function repair/transcription factor II H (TFIIH) and result in a NER defect, which explains the photosensitivity observed. Amongst the remaining 50 % of non-photosensitive TTD cases, a minority (about 10 %) carry biallelic mutations in *TTDN1*, a ubiquitously expressed gene thought to play a role in the maintenance of the cell cycle [4–6]. **Cockayne syndrome** (Orpha number 191) is a multisystem disorder characterized by intellectual disability, microcephaly, severe growth failure, sensory impairment, peripheral neuropathy and cutaneous photosensitivity [7]. Various degrees of severity have been described, including: a prenatal-onset form known as cerebro-oculo-facio-skeletal (COFS) syndrome (Orpha number 1466), an early-onset or severe form (CS type II), a “classical” or moderate form (CS type I) and a mild or late-onset form (CS type III). The two major genes responsible for the disorder, *ERCC6(CSB)* and *ERCC8(CSA)* were identified in the mid 1990s [8, 9]. More recently, defects in either XPF endonuclease or in its partner ERCC1 have also been associated with CS [10]. COFS patients show mutations mainly in *ERCC6(CSB)* [11, 12], but also in *ERCC2(XPD)* [13], *ERCC5(XPG)* [14–16] and *ERCC1* [17]. A few rare cases show combined features of **CS and XP**. Some of these patients present in the neonatal period with severe features such as in COFS, which leads to early mortality. Others have later onset and milder neurological/developmental abnormalities with XP-like skin lesions which develop in later life [18–20]. These combined presentations are mainly linked to mutations in *ERCC2(XPD), ERCC3(XPB)* or *ERCC5(XPG)* [16, 18, 19], but defects in *ERCC4* have also recently been observed [10]. Finally, **UV-sensitive syndrome** (Orpha number 178338) is characterized by isolated cutaneous photosensitivity without any of the other features associated with CS, and without the pre-disposition to skin malignancy as in xeroderma pigmentosum. It has been linked to mutations in *ERCC6(CSB),ERCC8(CSA)* and in the recently identified gene encoding for UV-stimulated scaffold protein A (*UVSSA*) [21–23]. Distinct functions of the CS proteins aside from their role in TC-NER, may account for the various clinical symptoms of CS and for the differences between CS and UVSS [24]. Intriguingly, neither the site nor the nature of the mutation in *CSA* or *CSB* seems to correlate with the clinical differences observed amongst patients with CS and UVSS [25].

**Table 1:** Clinical symptoms of NER-related disorders and more frequently involved genes

	Cockayne syndrome	COFS syndrome	Trichothiodystrophy	Xeroderma pigmentosum	UV-sensitive syndrome
Growth failure	++	++	+	-	-
Microcephaly	++	++	+/−	+/−	-
Intellectual disability	++	++	+/−	+/−	-
Retinal degeneration	+	+/−	-	-	-
Cataracts	+	+	+	-	-
Deafness	+	+/−	+/−	-	-
Photosensitivity	+	+/−	+/−	++	++
Brittle hair	-	-	++	-	-
Cancer	-	-	-	++	-
Known involved genes	*ERCC6(CSB)*	*ERCC6(CSB)*	*ERCC2(XPD)*	*XPC*	*ERCC6(CSB)*
	*ERCC8(CSA)*	*ERCC2(XPD)*	*ERCC3(XPB)*	*ERCC2(XPD)*	*ERCC8(CSA)*
		*ERCC5(XPG)*	*TTD-A*	*XPA*	*UVSSA(KIAA1530)*
		*ERCC1*	*TTDN1*	*POLH*	
				*ERCC5(XPG)*	
				*DDB2(XPE)*	
				*ERCC3(XPB)*	
				*ERCC1*	
				*ERCC4(XPF)*	

COFS: cerebro-oculo-facio-skeletal syndrome

Clinical recognition of NER defects remains challenging, due to the remarkable heterogeneity and overlap of clinical symptoms which exists between these conditions (Table 1). Moreover, the existence of combined forms, such as XP/CS, further complicates the diagnosis. Historically, the diagnosis was confirmed by DNA repair activity testing on primary fibroblasts, using ‘unscheduled DNA synthesis (UDS)’ to test GG-NER, and ‘recovery of RNA synthesis (RRS)’ to test TC-NER [26]. More recently, gene identification has permitted molecular diagnosis of NER related disorders, using Sanger sequencing of candidate genes. The clinical presentations, as well as the results of UDS and RRS cellular tests, are used to guide the investigations towards the gene involved. However, this gene-by-gene sequential approach is expensive and time consuming. Our goal was to develop a single and efficient mutation-screening strategy for all NER related disorders, and to improve our understanding of the clinical spectrum observed. In this study, we describe our findings, based on the enrichment of 16 genes of the NER pathway by multiplex amplification coupled with next-generation sequencing (NGS). Our cohort included 40 patients referred for suspicion of NER defects.

## Methods

### Patients and samples

Initially, the NGS procedure was tested and validated on a small cohort of 11 patients who had already been screened for the entire coding sequence of *ERCC6(CSB)* and/or *ERCC8(CSA)* genes. Next, a prospective cohort of 40 consecutive patients referred to our lab with suspicion of NER defects was studied. For all patients, written consent for genetic testing was obtained, either from adult probands or from a legal representative in the case of minors.

### DNA sample and quality control

Genomic DNA was extracted either from peripheral blood or fibroblast cultures following standard procedures (QIAGEN). DNA concentration and quality were assessed at each quality-control step using either Nanodrop® 8000, Qubit® 2.0 fluorometer (Life technologies), or LabChip® GX (Caliper).

### Multiplex amplification

Targeted genomic regions covered exons from the major isoform of the 16 genes involved in the NER pathway (Table 2). Exon coordinates were extended to an additional 50 bp in flanking intronic sequences. Entire 5’ and 3’ untranslated regions were included for *ERCC6(CSB)* and *ERCC8(CSA)* genes only. Primer design for multiplex amplification was performed with Ion AmpliSeq Designer version 2.2 (reference IAD43922_95, Life Technologies). The overall targeted regions span 62 kb, amplified in 452 amplicons (length between 125 and 175 bp), and are divided into two pools.

**Table 2:** Genes included in the targeted next generation sequencing strategy

Official gene symbol	Legacy name	Ref Seq NM #	# Exons	Size of coding exons (bp)	Targeted region size (bp)	Theoretical coverage
*DDB1*		NM_001923.4	27	3423	6,150	94.67 %
*DDB2*	*XPE*	NM_000107.2	10	1284	2,268	99.03 %
*ERCC1*		NM_001983.3	10	894	2,102	99.57 %
*ERCC2*	*XPD*	NM_000400.3	23	2283	4,894	98.12 %
*ERCC3*	*XPB*	NM_000122.1	15	2349	3,864	99.48 %
*ERCC4*	*XPF*	NM_005236.2	11	2751	3,862	100.00 %
*ERCC5*	*XPG*	NM_000123.3	15	3561	5,076	100.00 %
*ERCC6*	*CSB*	NM_000124.3	21	4482	9,127	99.99 %
*ERCC8*	*CSA*	NM_000082.3	12	1191	3,256	98.65 %
*GTF2H5*	*TTD-A*	NM_207118.2	3	216	418	100.00 %
*MPLKIP*	*TTDN1*	NM_138701.3	2	540	742	100.00 %
*POLH*		NM_006502.2	11	2142	3,152	100.00 %
*USP7*		NM_003470.2	31	3309	6,440	93.88 %
*UVSSA*	*KIAA1530*	NM_020894.2	14	2130	3,443	99.07 %
*XPA*		NM_000380.3	6	822	1,428	100.00 %
*XPC*		NM_004628.4	16	2823	4,767	100.00 %
		Total	227	34200	58,721	

Targeted genomic regions covered coding exons with their intron boundaries (50 pb) from the major isoform of 16 genes involved in NER pathway. 5’ and 3’ untranslated regions (UTR) were included for *ERCC6(CSB)* and *ERCC8(CSA)* genes only. The total panel size was 62 kb considering the 3 genes located on sex chromosomes (*SRY, AMELX* and *AMELY*) added as gender internal quality control

### NGS sequencing

NGS library preparation for sequencing of the targeted genes was performed by multiplex amplification using the Ion AmpliSeq Library Kit 2.0 (Life Technologies). Individual samples were then barcoded using Ion Xpress™ Barcode Adapters 1–16 Kits. The amplified librairies were purified using Agencourt AMPure XP Beads (Beckman Coulter). Before library pooling of up to 8 patients, amplified libraries were validated using the 2100 BioAnalyzer System (Agilent). Emulsion PCR of the pooled library was performed using the OneTouch™ 2 system (Life Technologies). Enrichment of the template-positive ion sphere particles (ISPs, containing clonally amplified DNA) was performed using the Ion OneTouch ES (Life Technologies), according to the manufacturer’s instructions. The percentage of templated ISPs compared to total ISPs was estimated using an Ion Sphere™ Quality Control kit on a Qubit® 2.0 Fluorometer (Life Technologies). The enriched ISPs were loaded on either Ion 314 (validation phase only) or Ion 316 chips and sequenced with an Ion Personal Genome Machine (PGM, Life Technologies).

### NGS bioinformatic analysis: read mapping, variant calling and filtering

Sequencing data was collected and analyzed by the Torrent Suite v4.4 (Life Technologies), including base calling, barcode sorting, alignement to the reference genome (GRCh37), variant calling and coverage analysis. Low stringency default parameters for germline mutations were used on the Variant Caller plugin of the Ion Torrent Browser with the exception of indel and multiple nucleotide polymorphism maximum strand bias that was set to 1.

DNA sequences were visualized using Alamut Visual 2.7.1 (Interactive Biosoftware). Initial variant filtering included the removal of frequent variants (minor allele frequency ≥ 0.01) present either in dbSNP142 [27], in the Exome Variant Server [28] or in the Exome Aggregation Consortium (ExAC) [29]. Recurrent variations found in more than 10 % of the patients in our cohort were also filtered. Subsequent variant annotation and ranking was performed using VaRank v1.4.0 [30] configured with Alamut Batch (Interactive biosoftware).

### Gap filling and variant confirmation using Sanger sequencing

Gaps in NGS coverage of the targeted regions (i. e. coverage <30X, the threshold recommended in the literature [31, 32]), were filled with Sanger sequencing for coding sequences of the *ERCC6(CSB)* and *ERCC8(CSA)* genes only. Variants identified by the NGS approach were confirmed by Sanger DNA sequencing and familial segregation analyses were performed as extensively as possible. Sequences were obtained either on 3130xl or 3500 Genetic Analyzer (Applied Biosystems), aligned with the Sequencing Pilot software (JSI) and compared with the corresponding genomic DNA reference sequence (GRCh37). Splicing variants were validated by Sanger sequencing on cDNA obtained by reverse transcription of RNA extracted from patient’s fibroblasts when available. All primer sequences are available upon request.

### Illumina OminiExpress-24 chips

Samples were processed on Illumina OmniExpress-24 v1 arrays using the Infinium assay as previously described [33], and results were analyzed using Illumina GenomeStudio software. Reference standards were set with the Illumina Genetrain 2.0 algorithm using the 96 samples processed during the same run. Regions of homozygosity were called with the Illumina cnvPartition 3.1.6 algorithm, with a minimal region size of 1 Mb.

### Cell lines and culture conditions

All cells used in our study were human primary fibroblasts derived from patients. Cells were cultured in Dulbecco’s Modified Eagle Medium (DMEM) (Ref. 21885–025, GIBCO) supplemented with 10 % FBS (Ref. 10500–064, GIBCO) and 1 % Antiseptic-Antimycotic (Ref. 15240–062, GIBCO) at 37 °C in a humidified atmosphere containing 5 % CO_2_ until RRS and UDS was initiated.

### RRS assay

Cells were plated on coverslips in 6-well plates at a confluency of 7x10^4^ cells per well. After 2 days of growing, cells were washed with PBS, followed by irradiation with a range of UV-C doses (6-12-20 J/m^2^). The non-irradiated plate(s) acted as references. After UV-irradiation, cells were incubated for 23 h for RNA Synthesis recovery in DMEM supplemented with FBS. Then, after washing with PBS, cells were labelled with 5-ethynyl-uridine (EU; Invitrogen) for 2 h. Cells were then washed again with PBS, followed by fixation and permeabilization. The last step involved an azide-coupling reaction and DAPI staining (Click-iT RNA HCS Assay, Invitrogen). Finally coverslips were washed in PBS, and mounted on glass slides with Ibidi Mounting Medium (Biovalley).

Photographs of the cells were taken with a fluorescent microscope (Imager.Z2) equipped with a CCD camera (AxioCam, Zeiss). The images were processed and analyzed with the ImageJ software. At least 50 cells were randomly selected, and the average nuclear fluorescent intensity was calculated.

### UDS assay

Cells were plated on coverslips in 6-well plates at a confluency of 7x10^4^ cells per well. After 2 days of growing, cells were washed with PBS, followed by irradiation with a range of UV-C doses (5-10-15 J/m^2^), the non-irradiated plate(s) being the reference. After UV-irradiation, cells were incubated for 3 h with 5-ethynyl-2’-deoxyuridine (EdU; Invitrogen) contained in F 10 medium without thymidine supplemented with dialyzed FBS and 5-fluoro-2-deoxyuridine (Fudr; Sigma). After washing with PBS, cells were then incubated for 15 min in full normal medium (F 10 + antibiotics + 15 % FBS) complemented with cold thymidine (thymidine 5’ triphosphate; Sigma). Cells were then washed again with PBS, followed by fixation and permeabilization, azide-coupling reaction and DAPI staining. At the end, coverslips were washed in PBS, and mounted on glass slides with Ibidi Mounting Medium (Ref. 50001, Biovalley).

Photographs of the cells were taken and analyzed as for the RRS assay. At least 50 non-S-phase cells were randomly selected, and the average nuclear fluorescent intensity was calculated.

## Results

### Targeted regions: design strategy

Our goal was to develop an efficient mutation-screening process for the diagnosis of patients with phenotypes suggestive of NER defects. We chose a multiplex amplification approach coupled with NGS to focus on genomic sequencing of all NER genes known to be involved in human diseases at the time the study was designed, as well as on their direct interactors (Table 2). Three genes located on sex chromosomes (*SRY, AMELX* and *AMELY*) were included for quality control purposes.

### Analysis and evaluation

The procedure was first performed on a cohort of 11 samples, previously tested by Sanger sequencing of *ERCC6(CSB)* and/or *ERCC8(CSA)* genes. Pathogenic mutations were detected in 5 samples, as well as nonpathogenic variants which were found in all.

Initially, we tested several combinations using between one and four patient DNA samples, pooled together on 314 or 316 arrays. Based on these preliminary results, we then pooled 8 patient DNA samples on a 316 array for diagnostic testing.

We assessed the sensitivity and specificity of single nucleotide variation (SNV) detection by comparing NGS results with allelic states of SNVs detected by Sanger sequencing of *ERCC6(CSB)* and *ERCC8(CSA)* in the 11 samples. Several variation types (missense, nonsense, splice mutations and small deletion of one base) at different allelic states were tested (Additional file 1). All 63 previously identified single base variations were detected in their correct heterozygous/homozygous state. A further 94,321 normal *ERCC6(CSB)* and/or *ERCC8(CSA)* bases were also correctly sequenced with no false positives. In our sample (*N* = 63 variations, with 100 % correct detection rate), the probability of a false-negative event can be estimated at 3/N (=5 % with *N* = 64). This corresponds to an overall sensitivity of ≥95 %, calculated using the ‘rule of 3’ estimate of power, with respect to sample size [34]. The overall specificity, calculated by the same method using data from the 94,321 normal bases, can be estimated at ≥99.99 %.

Finally, analysis of variations in copy number was assessed using an amplicon-based enrichment NGS technique, a quantitative method used to compare the number of reads for each amplicon in each sample of the run [35]. A heterozygous 4.6 Mb deletion of chromosome 10q11, encompassing the whole *ERCC6(CSB)* gene, was clearly detected by this process, as well as by direct observation of the coverage data using Alamut Visual. In contrast, we were unable to detect an *ERCC6(CSB)* deletion limited to the first two exons of the gene, with this technique (data not shown).

### Results from the 40 patient cohort

We analyzed data obtained from a cohort of 40 consecutive patients referred for suspected Cockayne syndrome (*n* = 30), xeroderma pigmentosum (*n* = 5), UV sensitive syndrome (*n* = 2) or COFS syndrome (*n* = 3). Our technique allowed us to generate a high-quality sequencing dataset, with a mean depth of coverage of 944X. On average, 95 ± 3 % of targeted regions were covered more than 30X for each patient. Depth of coverage was highly variable (944 ± 493 X) but always over 172X. Few regions, (*n* = 14 out of 452 amplicons, or 3 % of the amplicons) appeared to be consistently or frequently (>50 % of the patients) poorly covered (mean coverage < 30X) (Additional file 2). Of these, the first group are mainly highly GC-rich regions which are difficult to amplify, and also difficult to study by capture enrichment processes as demonstrated by the limited coverage of these regions on the ExAC database. Other poorly covered regions are low GC-content regions with numerous small polyA or polyT tracts. Such homopolymer stretches are well known to be prone to high error rates with Ion Torrent PGM sequencing technology [36, 37]. An average of 67 ± 12 unfiltered variations per patient was observed.

We detected clear pathogenic biallelic mutations in 17 of the 40 cases (43 %). No potentially pathogenic variant could be identified in any of the 16 targeted genes in the remaining 23 patients (57 %).

### Diagnosis of patients with classical phenotypes

Amongst the confirmed mutation group (17 patients), two thirds (*n* = 11) displayed a molecular diagnosis corresponding to the classical clinical presentation. Concerning CS, four patients (# 1 to 4) showed biallelic mutations in the *ERCC6(CSB)* gene and three patients (# 5 to 7) in the *ERCC8(CSA)* gene. They all display a classical Cockayne phenotype with growth failure, microcephaly, intellectual disability, pyramidal and extra-pyramidal signs and neurosensorial impairment (Table 3). Several novel mutations were found in these two genes including 2 frameshift, 3 splicing and 1 missense mutation (Table 4). None of these variants were present in the EVS or ExAC databases. The novel *ERCC8(CSA)* missense variant, c.356C > T p.Ser119Leu, involved an amino-acid located in a WD40 repeat domain. It is predicted to be deleterious by SIFT, probably damaging by PolyPhen2 and disease causing by MutationTaster. Whilst the parents were not available for segregation analysis (patient #6), this missense mutation is located in a region in which many other clearly pathogenic missenses have been reported. The second mutation in our patient is a splice consensus mutation with a high pathogenicity potential.

**Table 3:** Clinical description, molecular and cellular results of the 40 NER-defective patients studied by NGS

Patient #	Gender	Clinical indication	Intellectual disability/psychomotor delay	Growth failure	Micro-cephaly	Photo-sensitivity	Neuro-sensorial impairement	Cancer	RRS	UDS	NGS diagnosis	Mutated gene	Mutations (c.)	Mutations (p.)	Parentation segregation
1	F	CS	+	+	+	+	+	-	↓	NA	+	*ERCC6(CSB)*	c.[(?_-164)_(−15+44+1_136-1)];[2047C>T]	p.[?];[Arg683*]	+ (only mother)
2	M	CS	+	+	+	+	+	-	↓	NA	+	*ERCC6(CSB)*	c.[2599-26A>G];[**4115delG**]	p.[Met867Thrfs*14];[Gly1372Glufs*22]	+
3	M	CS	+	+	+	NA	+	-	NA	NA	+	*ERCC6(CSB)*	c.[2060C>T];[3862C>T]	p.[Ser687Leu];[Arg1288*]	+
4	M	CS	+	+	NA	+	-	-	↓	N	+	*ERCC6(CSB)*	c.[**543G>T**];[543+4delA]	p.[**Lys181Asn**];[?]	+
5	F	CS	+	+	+	+	+	-	NA	NA	+	*ERCC8(CSA)*	c.[**927delT**];[1041+1G>T]	p.[Phe309Leufs*19];[?]	**+**
6	F	CS	+	+	+	++	+	-	↓	N	+	*ERCC8(CSA)*	c.[**356C>T**(;)618-1G>A]	p.[**Ser119Leu**(;)?]	NA
7	M	CS	+	+	+	+	-	-	↓	NA	+	*ERCC8(CSA)*	c.[611C>A];[**1122+1delG**]	p.[Thr604Lys];[?]	+ (only mother)
8	M	CS	+	-	-	+	+	-	I	N	+	*ERCC8(CSA)*	**c.[730C>T];[730C>T]**	**p.[His244Tyr];[His244Tyr]**	+
9	M	CS	+	+	+	+	+	-	I	NA	+	*ERCC8(CSA)*	**c.[793A>C];[793A>C]**	**p.[Thr265Pro];[Thr265Pro]**	**+**
10	F	XP	-	NA	-	+	-	+	N	N	+	*POLH*	**c.[764+1G>A(;)1445C>A]**	p.[?(;)Ser482*]	NA
11	F	XP	-	NA	NA	+	-	+	N	N	+	*POLH*	**c.[764+1G>A(;)764+1G>A]**	**p.[?(;)?]**	NA
12	F	XP	-	NA	-	+	-	+	N	N	+	*POLH*	**c.[764+1G>A(;)764+1G>A]**	**p.[?(;)?]**	NA
13	M	XP	-	NA	NA	+	NA	+	N	N	+	*POLH*	**c.[764+1G>A];[764+1G>A]**	**p.[?];[?]**	+
14	M	CS	+	-	+	++	+	-	NA	↓	+	*ERCC2(XPD)*	c.[1847G>C];[2047C>T]	p.[Arg616Pro];[Arg683Trp]	+ (only mother)
15	F	UVSS	-	-	-	++	-	-	NA	↓	+	*ERCC2(XPD)*	c.[2047C>T];[2047C>T]	p.[Arg683Trp];[Arg683Trp]	+
16	F	CS	+	+	+	+	-	-	↓	↓	+	*ERCC5(XPG)*	**c.[2200-10C>G];[2200-10C>G]**	**p.[?];[?]**	**+**
17	M	UVSS	-	-	-	++	+	-	↓	↓	+	*ERCC3(XPB)*	c.[296T>C(;)**325C>T**]	p.[Phe99Ser(;)Arg109*]	NA
18	M	CS	+	+	+	-	+	-	NA	NA	-				
19	F	CS	+	+	+	NA	+	-	N	↓	-				
20	F	XP	+	+	NA	++	+	-	NA	NA	-				
21	M	CS	+	+	+	NA	-	-	N	N	-				
22	F	CS	+	+	+	-	+	-	I	N	-				
23	M	CS	+	+	+	NA	+	-	I	NA	-				
24	F	CS	+	+	-	-	-	-	N	N	-				
25	M	CS	+	+	+	-	-	-	N	NA	-				
26	F	CS	+	+	+	-	-	-	N	N	-				
27	M	CS	-	+	+	-	+	-	NA	NA	-				
28	M	COFS	NA	+	+	NA	+	-	N	N	-				
29	M	CS	+	+	+	+	+	-	NA	NA	-				
30	F	CS	+	+	+	+	+	-	N	↓	-				
31	M	CS	+	+	+	-	+	-	NA	NA	-				
32	F	CS	+	+	+	+	-	-	↓	N	-				
33	M	CS	+	+	+	-	-	-	N	N	-				
34	F	CS	+	+	+	-	-	-	NA	NA	-				
35	F	CS	+	+	+	-	+	-	NA	NA	-				
36	F	CS	+	+	+	-	+	-	NA	NA	-				
37	M	CS	+	+	+	-	+	-	NA	NA	-				
38	F	CS	+	+	+	NA	+	-	NA	NA	-				
39	F	COFS	NA	+	+	NA	NA	-	NA	NA	-				
40	M	COFS	NA	+	+	NA	NA	-	N	I	-				

Undescribed variations are in bold. Variations are described according to the latest nomenclature conventions described in HGVS. I: inconclusive results, N: normal; NA: not available; RRS: recovery of RNA synthesis; UDS: unscheduled DNA synthesis

**Table 4:** Novel variations identified in *ERCC6(CSB), ERCC8(CSA), ERCC2(XPD), ERCC3(XPB) ERCC5(XPG)* and *POLH* genes

Gene	DNA variant	cDNA variant	Protein	Type (DNA)	Localization	Variant predicted effect	Functionnal domain	Frequency in EVSdb	Frequency in ExACdb	Prediction among SIFT and PolyPhen 2 and Mutation Taster	Patient #
*ERCC6(CSB)*	c.543G>T	r.423_543del (exon 3 deletion)	p.Lys181Asn	Substitution	exon 3	Missense + splicing	-	NF	NF	D, PD, DC	4
	c.4115delG	-	p.Gly1372Glufs*22	Deletion	exon 21	Frameshift	-	NF	NF	-	2
*ERCC8(CSA)*	c.356C>T	-	p.Ser119Leu	Substitution	exon 4	Missense	WD40	NF	NF	D, PD, DC	6
	c.730C>T	-	p.His244Tyr	Substitution	exon 9	Missense	WD40	NF	NF	D, PD, DC	8
	c.793A>C	-	p.Thr265Pro	Substitution	exon 9	Missense	WD40	NF	NF	D, PD, DC	9
	c.927delT	-	p.Phe309Leufs*19	Deletion	exon 10	Frameshift	-	NF	NF	-	5
	c.1041+1G>T	Not tested	p.?	Substitution	intron 10	Splicing	-	NF	NF	-	5
	c.1122+1delG	Not tested	p.?	Deletion	intron 11	Splicing	-	NF	NF	-	7
*ERCC3(XPB)*	c.325C>T	-	p.Arg109*	Substitution	exon 3	Nonsense	-	0.000462	0.000478	-	17
*ERCC3(XPB)*	c.2200-10C>G	r.2199_2200ins2200-9_2200-1 (partial insertion of intron 9)	p.Glu734_Thr1186delinsIleLeu*	Substitution	intron 9	Splicing	-	NF	NF	-	16
*POLH*	c.764+1G>A	r.723_764del42 (partial del exon 6)	p.Ser242_Ile255del14	Substitution	intron 6	Splicing	-	NF	8.241e-06	-	10, 11, 12, 13
			p.Val221Profs*2								
		r.661_764del104 (del exon 6)									
*POLH*	c.1445C>A	-	p.Ser482*	Substitution	exon 11	Nonsense	-	NF	NF	-	10

*D* deleterious, *DC* disease causing, *EVSdb* Exome Variant Server database, *ExACdb* Exome Aggregation Consortium database, *NF* no frequency data available, *PD* probably damaging

Concerning XP, analysis of a group of six patients from four families coming from Northern Spain (Cantabria, *n* = 1, Basque Country, *n* = 5) allowed us to identify a founder mutation in the *POLH* gene (Additional file 2). All patients except for one have developed one or several skin cancers (melanomas, basocellular carcinomas and epidermoid carcinomas) with an onset of between 15 and 41 years of age. Four of the patients experience photophobia. Increased number of lentigines (*n* = 2/6) and exaggerated and prolonged sunburn response (*n* = 2/6) are also described. One patient suffers from hearing problems. No other symptoms have been reported. All patients exhibited a normal NER level after UV irradiation, even on UDS or RRS assay. This is a hallmark of XP variant (XP-V), a disease linked to the *POLH* gene. NGS was performed on a single patient from each family (patients # 10 to 13). In 3 of these 4 patients, we identified a homozygous *POLH* variation at the canonical donor site of intron 6, c.764 + 1G > A. Parental segregation confirmed the heterozygous carrier status of the parents in one family. The last patient (patient # 10), was compound heterozygous for the same *POLH* splicing mutation and for a novel *POLH* stop mutation: c.1445C > A p.Ser482*. cDNA studies on the homozygous patients revealed two truncated transcripts (Additional file 3): the first splice form, with the greatest expression, uses a cryptic splice donor site, 42 bp upstream from the end of exon 6. The resulting in-frame deletion (r.723_764del42; p.Ser242_Ile255del14) leads to shortening of the polymerase eta protein by 14 amino acids. The second splice variant has a deletion of the entire exon 6 (104 bp) (r.661_764del104) expected to produce a 221-amino-acid truncated protein (p.Val221Profs*2). Whereas the c.764 + 1G > A mutation is largely unknown (described once in the ExAC database, in one out of 11,564 alleles from a Latino population), the c.764 + 1G > C mutation has been described in patient XP31BE [38] with exactly the same abnormal transcripts. As expected in XP-V cell lines, fibroblasts collected from our Spanish patient exhibited a specific reduction in post-UV survival in the presence of caffeine (data not shown). Haplotype analysis using the Illumina HumanOmniExpress-24 SNP chip showed a common homozygous haplotype of 975 kb encompassing the *POLH* gene in the patients of families # 11, 12 and 13 (Additional file 4). The patient with compound heterozygosity presented a genotype over the 257 SNPs in the interval completely compatible with the founder haplotype.

### Uncommon diagnoses established by targeted NGS

The remaining third of cases from our confirmed mutation group (*n* = 6 out of 17 patients) displayed atypical findings such as mild and incomplete symptoms, combined phenotypes or involvement of extremely rarely implicated genes.

Of these cases, we identified two close *ERCC8(CSA)* missense mutations in two CS patients showing incomplete or mild symptoms. The younger patient (#8) is a 16 year old boy presenting with an incomplete clinical picture with normal growth (25th centile), slight intellectual deficiency, behavioral difficulties, congenital unilateral deafness, progressive enophthalmia and cutaneous photosensitivity (Additional file 5). The older patient (#9), a 26-year-old man, was referred for small stature, microcephaly, congenital deafness, learning difficulties and photosensitivity associated with truncal cutaneous spots and cerebellar atrophy (Additional file 5). Both patients, born to consanguineous parents, are homozygous carriers of a missense variant, respectively c.730C > T p.His244Tyr for patient #8 and c.793A > C p.Thr265Pro for patient #9. These variations, which are absent from the EVS and ExAC databases, involve conserved amino-acids localized in the same functional WD40 domain and are predicted as deleterious by Sift and PolyPhen2. Segregation studies have confirmed the carrier status of the parents in both families. The two healthy siblings of patient #8 and four healthy siblings of patient #9 were either heterozygous carriers of the familial missense variant or homozygous for the wild type allele. Studies of *ERCC8(CSA)* mRNA in patient #9 found a normal-sized transcript with the homozygous p.Thr265Pro missense mutation. RRS functional assay was inconclusive in these two cases: repeated experiments showed variable results which prevented us from drawing any formal conclusions about the efficiency of TC-NER in these patients under experimental conditions (data not shown).

One patient referred for CS and one for UVSS were linked to the *ERCC2(XPD)* helicase gene, with a final diagnosis of XP/CS and XP respectively. Patient #14 was the first child of young unrelated healthy parents. His birth weight was 2760 g (10^th^ centile), length was 47.5 cm (5^th^ centile) and his head circumference was 33 cm (5th centile). He was referred for early and severe photosensitivity, learning difficulties, peripheral neuropathy and deafness. At the age of 14, he presented microcephaly (head circumference: 50.5 cm (below the 3^rd^ centile)) without growth failure (height: 162 cm (mean) and weight: 44.4 kg (25^th^ centile)). No evidence of calcification or leucodystrophy was revealed on brain imaging. No cutaneous malignancies were described at the age of 16. The patient has a younger sister (15 year old) who presents a less severe phenotype without microcephaly or neuropathy, but with learning disabilities and mild photosensitivity. Surprisingly, we identified two compound heterozygous missense variations (already described) in *ERCC2(XPD)*: c.1847G > C and c.2047C > T. These lead respectively to the protein changes p.Arg616Pro and p.Arg683Trp. The p.Arg683Trp mutation was inherited from the mother, the father was not tested. The affected sister shares the same genotype as patient #14. Both variations, involving highly conserved amino-acids and located in a helicase domain, have been previously reported in either XP or in TTD patients [39, 40]. Our patient shared the same genotype as the XP17PV patient presenting with XP and mild neurological abnormalities [40]. His first tumor appeared at 22 years of age. The second *ERCC2(XPD)* patient of the cohort (#15) is the first child of consanguineous healthy parents, referred by the dermatologist for UVSS. She presents severe photosensitivity with normal growth and psychomotor development. No cutaneous cancer was described at the age of 4. She was found to be homozygous for the common *ERCC2(XPD)* missense mutation, c. 2047C > T p. Arg683Trp, as was her affected younger sister. The parents are heterozygous carriers of the mutation. Both patients display a severely decreased UDS.

A further CS patient was diagnosed with combined XP/CS, linked to the rarely involved *ERCC5(XPG)* gene*.* Patient #16 was the first child of young healthy parents. No consanguinity was known but the parents came from very close villages. The patient was born at term. Her birth weight was 2700 g (25^th^ centile), length was 51 cm (mean) and her head circumference was 31 cm (below 3^rd^ centile). Apgar score was 10. The patient was evaluated at the age of 4 years and 4 months. At that time, she was 81 cm tall, weighed 7 kg, and had a head circumference of 35 cm (all well below the 1^st^ centile). She displayed delayed developmental milestones (she sat at the age of one year, walked when she was 2 years old and spoke single words at the age of 3.5). She exhibited an abnormal ataxic gait. She also had deep set eyes and signs of retinal pigmentary degeneration. Auditory assessment was normal. She had cutaneous photosensitivity associated with dry skin and numerous pigmented naevi. Her brain MRI showed de-myelination of the white matter (Additional file 6). Liver enzymes were slightly elevated (SGOT = 43 U/l; SGPT = 99U/l). Both RRS and UDS were severely decreased. We identified a novel homozygous *ERCC5(XPG)* variation at the acceptor site of intron 9, c.2200-10C > G, the first splicing mutation described in this gene. Both parents were found to be heterozygous carriers of the variant. cDNA studies revealed a significant biological impact with no normal transcript but with several aberrant species leading to early protein termination. The major transcript included the last 9 bp of intron 9 between exon 9 and 10, leading to a premature stop codon (p.Glu734_Thr1186delinsIleLeu*). As expected in *ERCC5(XPG)* XP/CS cell lines [41], the patient’s fibroblasts exhibited a specific reduction of post-UV survival in the presence of methylene blue (data not shown).

The last atypical result involved a teenager referred for UVSS who was found to carry mutations in the *ERCC3(XPB)* gene*.* To date, eight different mutations in nine patients from six families have been reported. Patient #17 displayed severe and early-onset photosensitivity with erythema and progressive lentigines on exposed areas (Additional file 7). No skin cancer was described at the age of 13. General health was good, with normal growth and psychomotor development. Moderate unilateral hypoacousia was described. The patient has a younger brother who suffers from less severe photosensitivity. As expected, both UDS and RRS were severely decreased. We identified two close heterozygous substitutions in *ERCC3(XPB):* c.296 T > C and c.325C > T, leading to the p.Phe99Ser missense mutation and to the p.Arg109* nonsense mutation respectively. As both mutations were never observed on the same NGS read (Additional file 7), we can consider that they are located in *trans*, even without analyzing parental samples (which are unavailable). The p.Phe99Ser missense mutation has been previously reported in association with the c.471 + 1G > A p.K157insTSDS* splicing mutation in two mild XP/CS siblings [42] and with the c.1273C > T p.Arg425* nonsense mutation in two XP siblings [43]. The p.Arg109* nonsense mutation has never been described in NER patients but is present in the EVS and ExAC database at a very low frequency (0.08 % in Europeans). This apparent discrepancy between the rarity of reported cases of *ERCC3(XPB)* mutations and the relatively high frequency of the p.Arg109* mutation in Europeans raises the question of possible fetal lethality when truncated mutations in this gene are present on both alleles. This hypothesis is consistent with the absence of such cases in the litterature.

## Discussion

Our aim was to develop a single and efficient mutation-screening process for NER defects and to improve our understanding of the clinical spectrum of these overlapping and sometimes combined disorders. We performed targeted next generation sequencing of 16 NER genes in a cohort of 40 consecutive patients presenting with phenotypes suggestive of DNA repair defects. Our findings confirm the efficiency of this process, which is able to simultaneously analyze 95 ± 3 % of targeted regions in 8 patients in a single run. Coverage is sufficiently sensitive (overall sensitivity ≥95 % in the preliminary cohort) to guarantee reliable detection of heterozygous variants and small indels. Whole gene deletion is also detectable, but the method is currently not sensitive enough (in our hands), to detect smaller heterozygous deletions (for example, a heterozygous deletion of the first two exons of the *ERCC6(CSB)* gene was not detected). This could be explained by the heterogeneity of the samples tested in one run (various sample types analyzed such as blood or fibroblast cultures, various DNA extraction methods used), as well as by the small number of patients per run, preventing normalization of depth of coverage on a large cohort. These limitations are unfortunately difficult to improve upon. Nevertheless, missing heterozygous CNV is less harmful in the context of exclusive autosomal recessive disorders than in dominant diseases. Presence of a CNV could indeed be considered when a single point mutation is found, then checked by a quantitative assay.

We identified causative mutations in 43 % of the cohort (17 out of 40 patients). This approach permits rapid confirmation of the molecular diagnosis for classical presentations of CS and XP. It has established the diagnosis in seven typical CS cases (patients # 1 to 7) with pathogenic mutations in either *ERCC6(CSB)* or *ERCC8(CSA),* and in four XP cases (patients # 10 to 13) with mutations in *POLH*. The technique is also reliable in cases with uncommon phenotypes (combined features or unusually mild symptoms or very rarely involved genes). For example, two of our patients initially referred for CS have a final diagnosis of XP-CS complex (patient #14 mutated in *ERCC2(XPD)* and patient #16 mutated in *ERCC5(XPG)*). Two further cases referred for UVSS had mutations in XP genes (patient #15 in *ERCC2(XPD)* and patient #17 in *ERCC3(XPB)*). These results clearly illustrate the challenge that clinical diagnosis of NER defects presents, and entirely justifies the multi-gene approach in molecular diagnosis. The diagnosis of NER diseases usually relies on established clinical criteria [7, 44, 45] but these criteria are often only fulfilled in patients at a relatively advanced stage of the disease. Clinical diagnosis is therefore difficult in young or late-onset cases that do not entirely fit the criteria. NGS is a particularly good alternative for confirming such cases at an early stage. The benefits of early diagnosis are well known in NER defects, for example, in order to prevent cutaneous cancer in XP and to delay sensory impairments and manage feeding difficulties in CS. Appropriate genetic counseling is beneficial for family members in all cases.

As expected, the majority of causative mutations identified in the CS patients were found to be located in the two main CS genes, *ERCC6(CSB)* (patients #1 to 4) and *ERCC8(CSA)* (patients #5 to 9). We detected eight novel mutations in these genes, four missenses predicted to be pathogenic, two frameshift mutations by single nucleotide deletions, and two mutations in splicing donor consensus sequences (Table 4). Including the last review [44] as well as recent case reports [46–51], the number of known mutations now rises to 86 for *ERCC6(CSB)* and 38 for *ERCC8(CSA)*.

We also describe the first splicing mutation in *ERCC5(XPG)* in an XP-CS patient (patient #16, homozygous for the c.2200-10C > G mutation). Biallelic mutations in the *ERCC5(XPG)* gene have previously been associated with XP (group G), CS, XP-CS and COFS [14, 16]. In the NER pathway, the XPG endonuclease performs a 3’ incision of the abnormal strand and stabilizes the basal transcription factor TFIIH. Patients have either truncating or point mutations that abolish TFIIH interaction, with truncating mutations being associated with the more severe phenotypes. So far, around 20 patients with a defect in the *ERCC5(XPG)* gene and 30 different disease-causing mutations have been described worldwide [14, 16, 52, 53]: 20 nucleotide substitutions (9 nonsenses and 11 missenses), 9 deletions of only 1 to 4 bases, 1 duplication and 1 large deletion [16]. We identified a homozygous splicing mutation without any expression of the normal transcript in cutaneous fibroblasts, leading to a severe XP-CS phenotype. The patient, initially referred for suspected CS, has a more complex diagnosis. This molecular finding radically changes the clinical management, which will include cutaneous cancer prevention and genetic counseling for the family.

The *ERCC3(XPB)* gene is even more rarely involved in human diseases. Coding a helicase, it is mutated in only nine reported patients from six families, with remarkable phenotypic heterogeneity: XP/CS (5 patients from 4 families), relatively mild XP without CS (2 siblings) and TTD (2 siblings) [43]. To date only eight different mutations have been identified in *ERCC3(XPB)*: 2 missense mutations, 4 truncating mutations (nonsense mutations, deletion or insertion of one base pair) and 2 splicing mutations [43]. Genotype/phenotype correlation studies of the short *ERCC3(XPB)* patient cohort have suggested that partially active missense mutations may explain symptoms in milder patients, whilst severe XP/CS complex patients have nonsense or consensus splice mutations with low levels of altered XPB proteins. Patient #17, a teenager referred for UVSS due to severe and early onset photosensitivity (without skin malignancy or neurological impairment) (Table 3), was found to be compound heterozygous for the known p.Phe99Ser mutation and for a novel nonsense p.Arg109* mutation. A similar genotype was found in two XP siblings, XP33BR and XP1SA, which carry p.Phe99Ser and p.Arg425* mutations [43]. *ERCC3(XPB)* mutations have been reported in XP/CS, XP alone and TTD patients, but never in UVSS patients as of yet. As our patient is only 13 years old, skin malignancy may still develop. Patients XP33BR and XP1SA which carry a similar genotype, both had multiple basal cell carcinomas, the first of which was diagnosed at 28 and 29 years old [43]. Long term follow up of our patient is necessary in order to test the hypothesis that the clinical spectrum of *ERCC3(XPB)* mutations extends to UVSS. As described previously, once again the molecular diagnosis leads to appropriate clinical follow-up.

Our cohort also included six XP patients from 4 unrelated families coming from the Basque country and surrounding area in Northern Spain. Surprisingly, all cases had mutations in the same gene, *POLH*, encoding the translesion DNA synthesis polymerase eta. Biallelic mutations in this gene have previously been associated with Xeroderma-Pigmentosum Variant (XP-V), which is distinct from classical XP due to the absence of insufficient DNA repair measured either by UDS or RRS. XP-V patients also have a relatively milder phenotype with late onset symptoms and delayed progression [54]. More than 60 mutations have been identified in the *POLH* gene, in cell lines derived from XP-V patients from different geographic locations [54]. The four patients from our cohort and their two siblings shared the same previously undescribed splicing mutation (NM_006502.2:c.764 + 1G > A). This was either in the homozygous form (5 out of 6 patients) or in combination with a novel nonsense mutation (NM_006502.2:c.1445C > A, p.Ser482*) (in one patient). Haplotype analysis showed a founder haplotype of 975 kb encompassing the *POLH* gene in the 4 families that were investigated (3 homozygous, 1 compound heterozygous). This suggests that a common ancestor carrying the mutation lived approximately 500–1000 years ago (using comparison calculations for a similar sized founder haplotype) [55]. Similar founder mutations in the *POLH* gene have been reported in other populations such as in Japan, Korea [38, 56] and Tunisia [57]. Such founder effects have previously been described in patients from the Basque country for several other diseases such as Parkinson disease [58], fatal familial insomnia [59] and limb-girdle muscular dystrophy type 2A [60]. These findings are consistent with the fact that this population has been relatively isolated in terms of genetic mixing in the past. Consequently, we suggest systematic screening for this mutation in all mild XP patients coming from the Basque country and its surrounding area.

Under our experimental conditions, classical functional assays failed to clearly identify two mild CSA patients (# 8 and 9), who have proven pathogenic mutations in the same WD40 proteic domain. We suspect that fluorescent RRS assay may overlook mild CS cases, especially when associated with missense mutations. A different patient carrying a homozygous missense mutation in the same domain was reported to have low RRS, but this was associated with a more severe type I clinical phenotype (patient 08STR2 [25]). In the few previously reported cases of mild late-onset CS, decreased RRS has always been an associated finding [25, 45, 61–69]. However, the classical diagnostic procedure for CS usually recommends performing molecular sequencing only after identification of a functional defect in the RRS, and thus atypical CS patients with normal or inconclusive RRS may be missed. We believe that these results should be included in the decision making tree for diagnosis of NER-diseases. In the flowchart proposed by Jia N et al., the authors suggest performing UDS and RRS assays prior to molecular sequencing (either by the Sanger method or NGS) [70]. Our experience suggests that strong clinical suspicion should prompt molecular investigations. We advocate performing cellular and molecular tests in parallel, as in most cases cellular tests still remain important for correct interpretation of nucleotide variants found by NGS.

Finally, no potentially pathogenic variants were identified in 23 patients included in the cohort (57 %). All except for one was referred for CS or COFS syndrome. Gap filling by Sanger sequencing of the two most commonly involved genes *ERCC6(CSB)* and *ERCC8(CSA)* did not reveal any anomalies*.* This relatively high rate of undiagnosed patients may be explained in part by the fact that 15 % of the cohort (6 out of 40 patients) had already been explored by Sanger sequencing of both *ERCC6(CSB)* and *ERCC8(CSA)* genes. Such “prescreening” artificially decreases the rate of positive diagnoses. Moreover, the 40 patients included in the cohort were not selected to fulfill the criteria of NER diseases precisely. Rather, they represent the day to day cases referred to our laboratory, with many patients presenting non-specific symptoms such as microcephaly or intellectual disability. Widening the inclusion criteria allows for diagnosis in patients who do not present all the classic diagnostic features, but it also leads to a lower rate of positive diagnoses overall. Most of these negative results occur in patients who are probably not true CS patients, and further molecular investigations are needed in order to establish a differential diagnosis. In one case (patient # 32), the phenotype was evocative of a DNA repair defect measured by cellular tests. This case highlights the limits of our targeted approach, as pathogenic mutations could be localized in non-targeted regions such as promoters or deep intronic regions. Such mutations are indeed known in the *ERCC8(CSA)* gene, with the example of the deep intronic c.173 + 1046A > G mutation [25]. While cDNA sequencing of *ERC6(CSB)* and *ERCC8(CSA)* genes did not retrieve any abnormality in patient #32, other NER transcripts have not been studied. Another explanation could be the presence of a small copy number variation (CNV), barely detectable by multiplex-based NGS strategies. Finally, a non-targeted gene could be involved in this patient. At least two new genes have been documented in NER disorders since our study was designed. A missense mutation in the proliferating cell nuclear antigen (*PCNA*), an essential DNA replication accessory protein, was identified in a single family with four patients sharing phenotypic similarities to XP, CS and also ataxia telangiectasia (AT) [71]. In cells from affected individuals, both UDS and RRS were reproducibly decreased. A family with a novel X-linked form of non-photosensitive TTD was also recently described, with a nonsense mutation in a gene of unknown function, *RNF113A* [72]. Whereas the targeted sequencing approach is the simplest tool for analyzing the genetic variants of a focused panel of genes via NGS, it will miss newly identified genes and prevents the re-analysis of data. Exome or full-genome strategies are more exhaustive alternatives, and are recommended in these patients. Such approaches have been succesfully used or suggested for the diagnosis of XP [73, 74].

## Conclusions

Targeted NGS of 16 genes involved in the NER pathway has been demonstrated to be an efficient alternative to the sequential Sanger approach for the molecular diagnosis of patients with a suspected NER defect, with a positive diagnosis rate of 43 %. We believe that this novel approach is the most appropriate solution to the diagnostic challenge presented by patients with combined specific neurological, dermatological and growth characteristics. This approach enables rapid confirmation of the molecular diagnosis in classical presentations of CS and XP. It can also be used to determine atypical phenotypes with combined features or very mild symptoms. Targeted NGS should be used in addition to DNA repair functional tests and precise clinical assessment in order to allow rapid, thorough and cost-effective diagnosis in patients with NER-defects.

## Additional files

Additional file 1: Spectrum of previously identified variations within the validation cohort of 11 patients. 11 patients already tested by Sanger sequencing of *ERCC6(CSB)* and/or *ERCC8(CSA)* genes were explored by targeted NGS strategy. All 63 previously identified single base variations were identified in their correct heterozygous/homozygous state. A heterozygous 4.6 Mb deletion of chromosome 10q11, encompassing the whole *ERCC6(CSB)* gene, was clearly detected but the method was not sensitive enough to detect an *ERCC6(CSB)* deletion limited to the first two exons of the gene. (XLSX 14 kb) Additional file 2: Poorly covered amplicons (mean coverage < 30X). Some amplicons appeared to be consistently poorly covered (in all patients of the cohort); others appeared frequently poorly covered (in more than 50 % of the patients) (XLSX 13 kb) Additional file 3: Novel *POLH* splice mutation in non-related XP-variant patients. A: Pedigrees of the 4 XP-variant families coming from Northern Spain and mutated in the *POLH* gene. The arrows indicate the four patients studied by NGS. Patients #11, 12 and 13 were homozygous for the splice mutation c.764 + 1G > A whereas patient #10 was compound heterozygous for mutations c.764 + 1G > A and c.1445C > A (p.Ser482*). B: RT-PCR using RNA from patients #10 and 11, with forward primer in exon 5 and reverse primer in exon 8, showed a single 365 bp band in normal cells and two bands at323 bp and 261 bp in the homozygous patient #11. The diagram indicates partial deletion (42 bp) of exon 6 in the type I splice variant and deletion of the entire 104 bp of exon 6 in the type-II splice variant. The normal transcript was the major transcript in the heterozygous patient #10, with a fain band corresponding to the type I splice variant. (PPTX 92 kb) Additional file 4:
*POLH* founder mutation in Northern Spain. Haplotype analysis was performed on the 6 XP-variant patients coming from Northern Spain with mutations in the *POLH* gene (chr6:43,543,878-43,588,260[hg19]). Despite a genome wide study using the Illumina HumanOmniExpress-24 SNP chip, the table focuses on the SNP localized in the vicinity of the *POLH* gene on chromosome 6 (between 42,000,000 and 44,000,000). Patient #11, patient #12 and her sister and patient #13 and his sister were homozygous for the c.764 + 1G > A mutation. Patient #10 was compound heterozygous c.[764 + 1G > A(;)1445C > A]. Parents of patient #13 were heterozygous carriers of the splice mutation. The five homozygous patients share a region of homozygosity (975 kb) including the *POLH* gene (from rs3800291 at position 42,751,457 to rs866236 at position 43,726,956). The compound heterozygous patient #10 and the carrier parents have at least one allele in common with the founder haplotype all along the 975 kb region. (XLSX 46 kb) Additional file 5: Clinical pictures of patients #8 (A) and #9 (B) (mutated in *ERCC8(CSA)*). (PPTX 403 kb) Additional file 6: Clinical pictures and brain MRI of patients #16 (mutated in *ERCC5(XPG)*). Sagittal T1 and axial T2-Flair (PPTX 970 kb) Additional file 7: Clinical picture and molecular results of patients #17 (mutated in *ERCC3(XPB)*). A: clinical picture of patient #17. B: Patient #17 reads alignment (thanks to Alamut Visual) showing that both mutations c.296 T > C and c.325C > T were never observed on the same read. Forward reads are in green and reverse reads are in blue. (PPTX 477 kb)
