# Supplementary figures and images for: Uncommon nucleotide excision repair phenotypes revealed by targeted high-throughput sequencing
Source: Orphanet J Rare Dis. 2016 Mar 22;11:26. doi: 10.1186/s13023-016-0408-0 (PMC4804614; doi:10.1186/s13023-016-0408-0)

## Slide 1
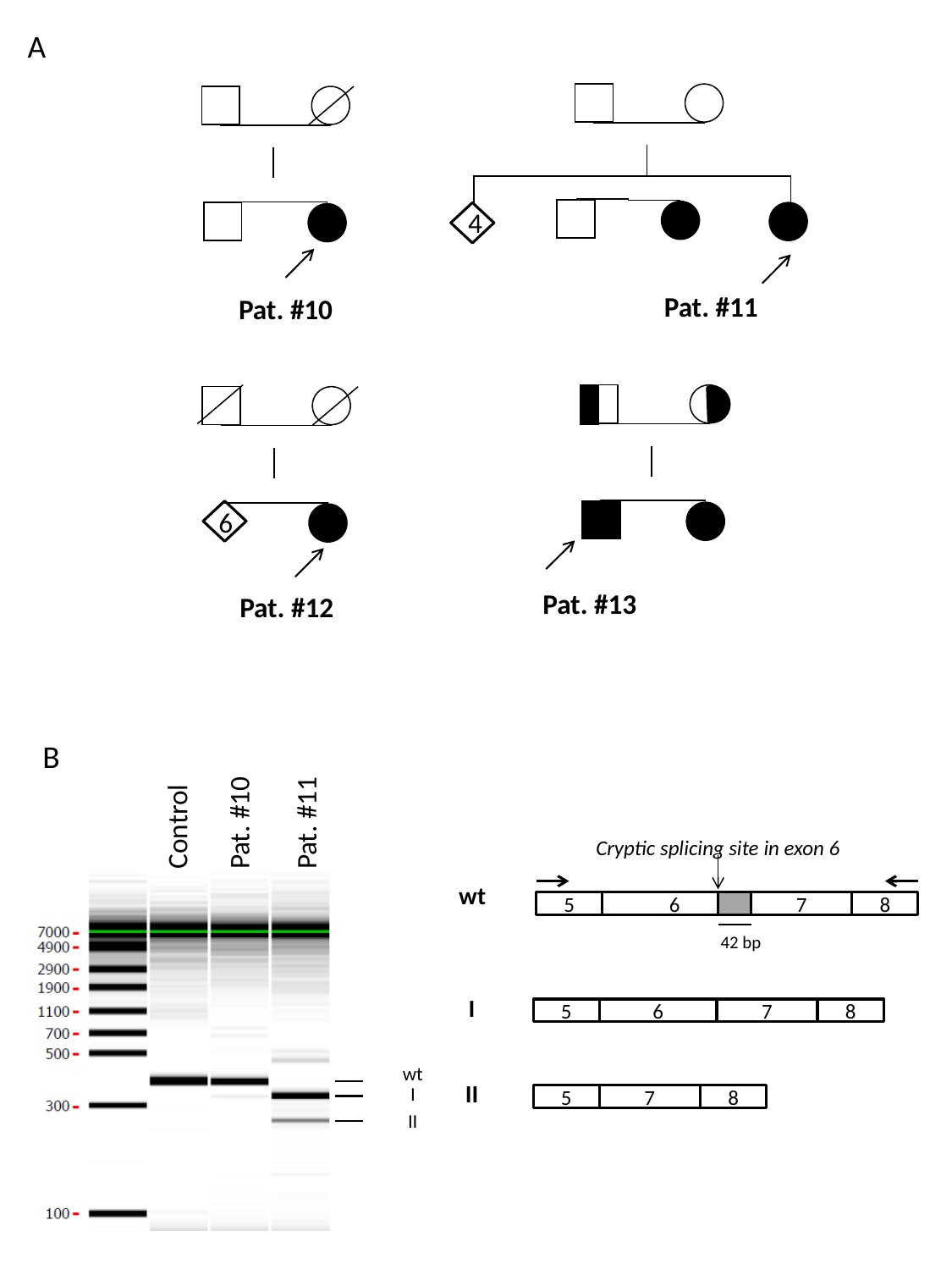

A
4
Pat. #11
Pat. #10
6
Pat. #12
Pat. #13
B
Pat. #10
Pat. #11
Control
wt
I
II
Cryptic splicing site in exon 6
5
6
7
8
42 bp
wt
I
5
6
7
8
II
5
7
8

Supplement: Additional file 3: — Novel POLH splice mutation in non-related XP-variant patients. A: Pedigrees of the 4 XP-variant families coming from Northern Spain and mutated in the POLH gene. The arrows indicate the four patients studied by NGS. Patients #11, 12 and 13 were homozygous for the splice mutation c.764 + 1G > A whereas patient #10 was compound heterozygous for mutations c.764 + 1G > A and c.1445C > A (p.Ser482*). B: RT-PCR using RNA from patients #10 and 11, with forward primer in exon 5 and reverse primer in exon 8, showed a single 365 bp band in normal cells and two bands at323 bp and 261 bp in the homozygous patient #11. The diagram indicates partial deletion (42 bp) of exon 6 in the type I splice variant and deletion of the entire 104 bp of exon 6 in the type-II splice variant. The normal transcript was the major transcript in the heterozygous patient #10, with a fain band corresponding to the type I splice variant. (PPTX 92 kb) [file 13023_2016_408_MOESM3_ESM.pptx]

## Slide 1
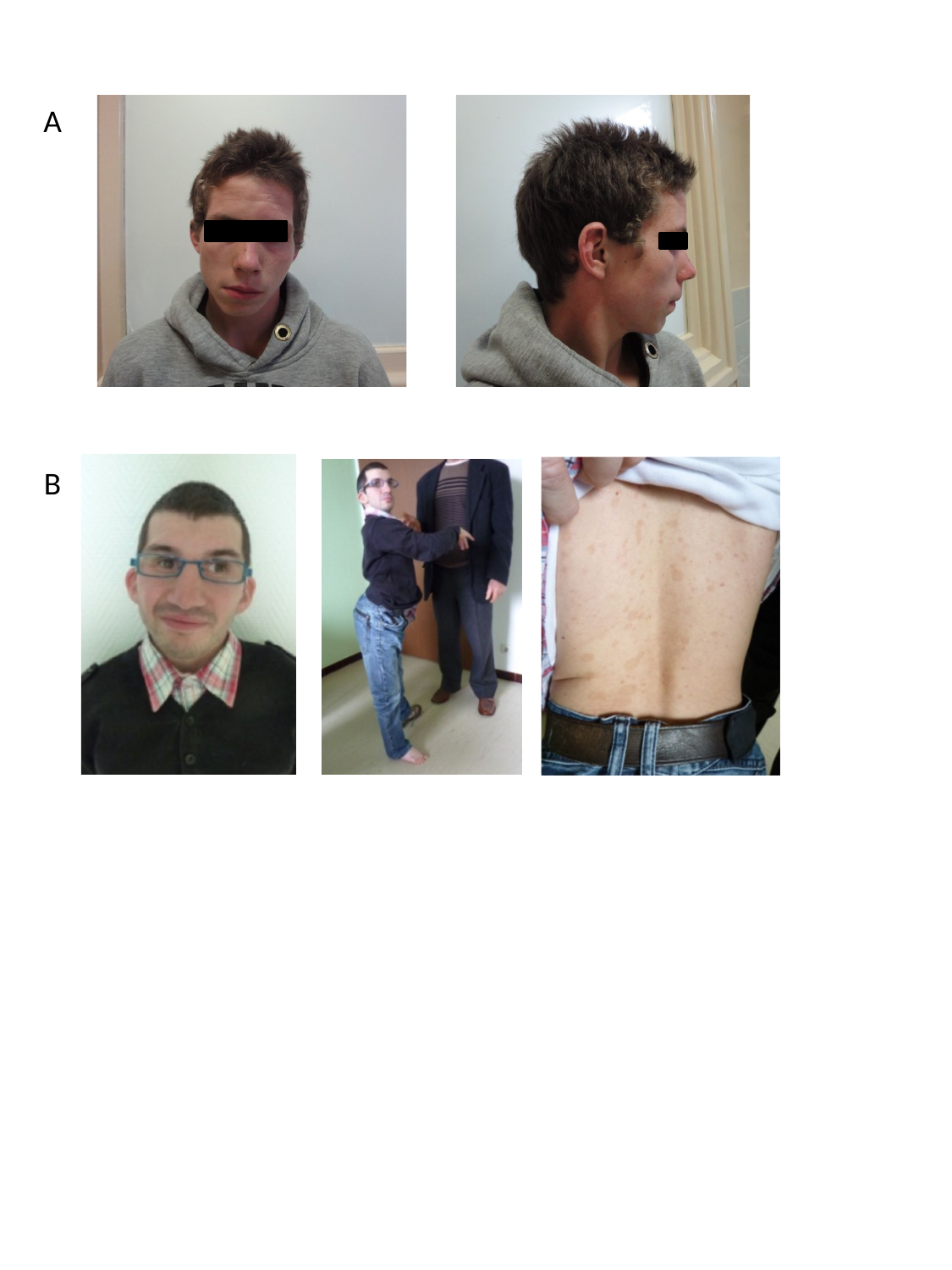

A
B

Supplement: Additional file 5: — Clinical pictures of patients #8 (A) and #9 (B) (mutated in ERCC8(CSA)). (PPTX 403 kb) [file 13023_2016_408_MOESM5_ESM.pptx]

## Slide 1
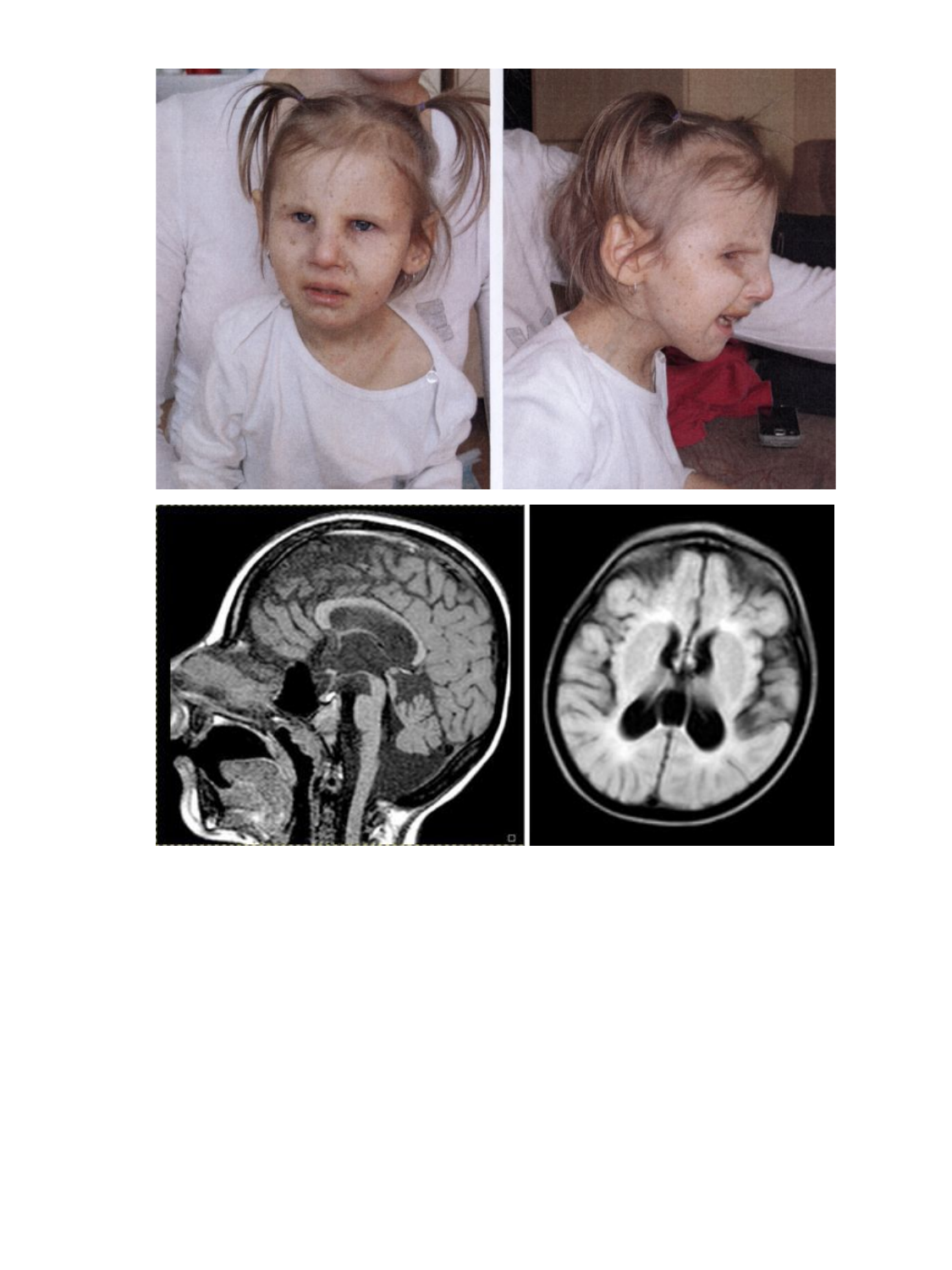

Supplement: Additional file 6: — Clinical pictures and brain MRI of patients #16 (mutated in ERCC5(XPG)). Sagittal T1 and axial T2-Flair (PPTX 970 kb) [file 13023_2016_408_MOESM6_ESM.pptx]

## Slide 1
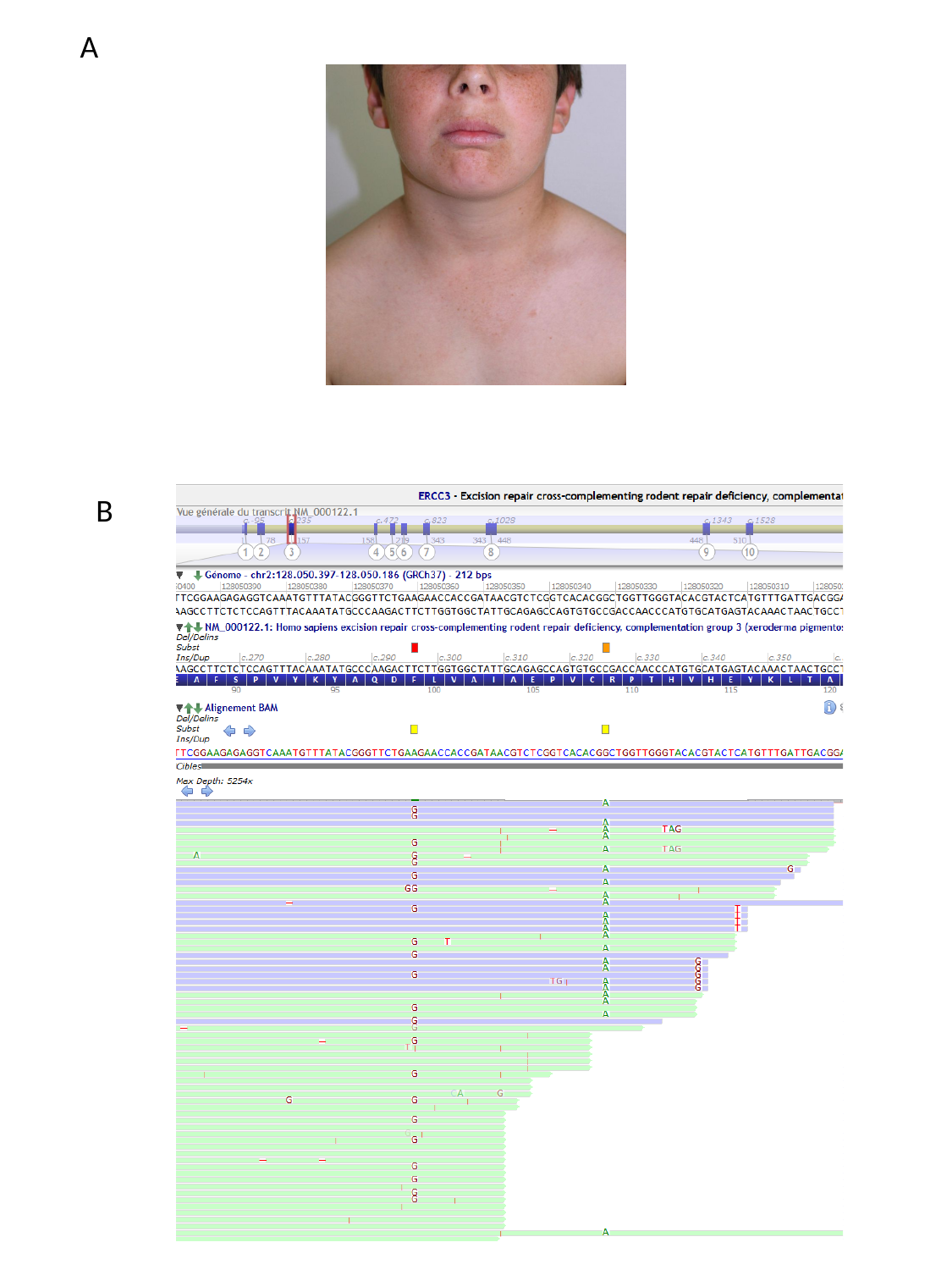

A
B

Supplement: Additional file 7: — Clinical picture and molecular results of patients #17 (mutated in ERCC3(XPB)). A: clinical picture of patient #17. B: Patient #17 reads alignment (thanks to Alamut Visual) showing that both mutations c.296 T > C and c.325C > T were never observed on the same read. Forward reads are in green and reverse reads are in blue. (PPTX 477 kb) [file 13023_2016_408_MOESM7_ESM.pptx]
